# Lunasin in cereal seeds: What is the origin?

**DOI:** 10.1016/j.jcs.2013.01.013

**Published:** 2013-05

**Authors:** Rowan A.C. Mitchell, Alison Lovegrove, Peter R. Shewry

**Affiliations:** a Department of Plant Biology and Crop Science, Rothamsted Research, Harpenden, Hertfordshire AL5 2JQ, UK; b School of Agriculture, Policy and Development, University of Reading, Earley Gate, Whiteknights Road, Reading RG6 6AR, UK

**Keywords:** Wheat, Cereals, Soybean, Lunasin

## Abstract

Lunasin is a peptide from soybean seeds which has been demonstrated to have anticancer properties. It has also been reported in cereal seeds: wheat, rye, barley and Triticale. However, extensive searches of transcriptome and DNA sequence databases for wheat and other cereals have failed to identify sequences encoding either the lunasin peptide or a precursor protein. This raises the question of the origin of the lunasin reported in cereal grain.

Lunasin is a peptide from soybean seeds (Jeong et al., 2003; Odani et al., 1987) which has been demonstrated to have anticancer properties (Hernández-Ledesma et al., 2009). It comprises 43 amino acid residues and is characterised by a high content of charged amino acids (19 in total), including a continuous sequence of nine aspartate residues at the *C*-terminus (sequence accession AAB71140). The characterisation of cDNAs encoding lunasin shows that it corresponds to the small subunit of the soybean 2S albumin (Galvez and de Lumen, 1999; Lin et al., 2004), with the large subunit having been previously characterised as an 8 kDa methionine-rich protein (Revilleza et al., 1996). The 2S albumins form a well-characterised family of seed storage proteins, which have been characterised from a wide range of dicotyledonous plants, with methionine-rich forms occurring in several species, notably Brazil nut and sunflower (Monsalve et al., 2003; Shewry and Pandya, 1999).

The biological activity of lunasin has led to searches for related peptides in other plant species, including reported isolation from *Solanum* (Jeong et al., 2007), amaranthus seeds (Maldonado-Cervantes et al., 2010; Silva-Sánchez et al., 2008) and cereal seeds: wheat (Jeong et al., 2007), rye (Jeong et al., 2009), barley (Jeong et al., 2002, 2010), and most recently Triticale (Nakurte et al., 2012). The identity of the peptides in barley and wheat was confirmed by partial sequences which match exactly to the soybean sequence over stretches of 22 and 14 amino acids, respectively (Fig. 1). This level of sequence identity is surprising considering the evolutionary distance between cereals and legumes and the fact that storage proteins diverge rapidly due to limited evolutionary constraints on their structures. It therefore implies a strong evolutionary pressure related to a precise function within the plant.

2S albumin storage proteins have not been reported in cereal seeds, although they are members of a wider superfamily of small sulphur-rich proteins which include non-specific lipid transfer proteins (LTPs), puroindolines (Pins) and inhibitors of α-amylase and trypsin present in cereal seeds. These proteins are also related to the prolamin storage proteins of wheat (gliadins and glutenins) and other cereals and are therefore often referred to as the “prolamin superfamily” (Shewry et al., 2004). We therefore decided to search available sequence databases using the sequence of the soybean peptide, in order to determine whether the lunasin identified in wheat and other cereals is also derived from a 2S albumin-like precursor.

1. The nr protein database available at NCBI (http://blast.ncbi.nlm.nih.gov/Blast.cgi) includes all non-redundant GenBank CDS translations + PDB + SwissProt + PIR + PRF totalling 58 million sequences, including 60,736 from the Triticeae. A search with the lunasin peptide (using blastp, *E*-value cut off < 0.1; low complexity filter off, other parameters set to default values; search conducted 9th October 2012) showed that the only sequences that exactly matched lunasin were from soybean, with the most closely related sequence from another species being from *Arachis hypogaea* (peanut), which is a legume within the same Papilionoideae subfamily as soybean. This sequence shares 60% identity with soybean lunasin over 25 amino acids, with the longest stretch of identity being four amino acids. This search would have found the wheat and barley sequences in Fig. 1 if they were present in the database, as the matches would have *E*-values of about 10^−6^ and 10^−15^, respectively. We then searched specifically within the 60,736 Triticeae sequences in this database at lower stringency (*E*-value < 10) to find the most similar sequence present in wheat and barley. The best matching sequences from wheat and barley were accessions ABR04074.1 and BAJ85510.1, respectively. The first of these is a HMW glutenin subunit 1By16 from *Triticum dicoccoides*, so could be a remote homologue within the prolamin superfamily. However, the alignments for these show only stretches of three or two identical amino acids and up to five similar amino acids (Fig. 2). These matches are many orders of magnitude less significant than those shown in Fig. 1 (probability of occurring by chance increased by about 10^8^ and 10^16^ for wheat and barley, respectively).
2. The Phytozome (www.phytozome.net) database includes the genomic sequences of all fully sequenced flowering plant genomes (27 species, including the cereals sorghum, maize and rice). A search for peptide sequences encoded by all predicted genes with the lunasin peptide (blastp, *E*-value cut off of 0.1; query filter off, other parameters set to default values; search conducted 3rd October 2012) showed a 100% match to one soybean gene which comprised a single exon, Glyma13g36400, indicating that this is the encoding gene. The only related gene that was identified in another species was again in a legume, *Medicago truncatula*, with no other hits being found at the same cut off. Although the barley genome is not yet present in Phytozome, a first draft of a comprehensive set of all genes has recently been released (International Barley Sequencing Consortium, 2012); tblastn searches of all 79,379 genes (26,159 ‘high confidence’, 53,220 ‘low confidence’ genes) revealed no better matches than that in Fig. 2B. Wheat has not yet been fully sequenced but lunasin-related sequences were also not found in the International Wheat Genome Sequencing Consortium chromosome sorted database of wheat genomic sequences (http://www.wheatgenome.org/).
3. EST (expressed sequence tags) sequence libraries comprise sequences derived from mRNAs (ie transcribed genes). The publically available libraries for barley and wheat total over 1.9 million ESTs from a wide selection of cultivars, with over half corresponding to mRNAs from seeds. Searching these and our own wheat RNA-Seq libraries from wheat starchy endosperm (12 million sequences) (Pellny et al., 2012) for transcripts encoding lunasin-like peptides (tblastn, *E*-value cut off of 0.1; query filter off, other parameters set to default values) identified no significant hits.

We also did not find matches for the lunasin sequence reported from the dicot amaranthus which shares 76% identity with soybean lunasin (Maldonado-Cervantes et al., 2010; Silva-Sánchez et al., 2008). However, this is not surprising as amaranthus species are very poorly represented in the databases (only 348 peptides in Genbank).

It is clear, therefore, that genes encoding peptides which are closely related in sequence to soybean lunasin are not present in the currently available sequence databases of wheat, barley or other major cereals. It is, of course, possible that genes including lunasin are present in cereals but are not represented in the databases. We think this is unlikely because the databases are extensive. Also, the reported levels of the lunasin peptide in cereal grains (reported as 14–21 μg/g and 12--99 μg/g in barley (Jeong et al., 2002, 2010), 429–6458 μg/g in triticale (Nakurte et al., 2012), 733–1510 μg/g in rye (Jeong et al., 2009) and 211–290 μg/g in wheat (Jeong et al., 2007)) indicate that significant levels of transcripts should be present, as in soybean and peanut where both genomic and transcript sequences were readily identified. This raises the question of the origin of the lunasin peptides reported by other workers in cereals (as discussed above). We have no explanation for this but one possibility is that the peptide is produced by microbiological action, as Rizzello et al. (2012) recently reported that the concentration of lunasin present after sourdough fermentation was 2–4 times greater than that present in control doughs. If so it must be assumed that the lunasin is synthesised and secreted by the microorganism, either as a mature peptide or a precursor which is then processed by a microbial or endogenous plant proteinase.

## Figures and Tables

**Fig. 1:**
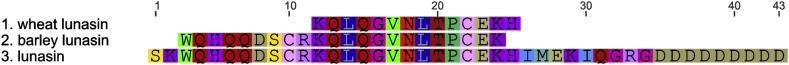
Alignment of peptide sequences reported for lunasins from wheat and barley seeds with the sequence of lunasin from soybean (based on sequences reported by Jeong et al., 2003, 2007, 2010).

**Fig. 2:**
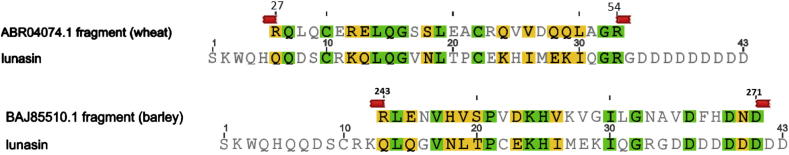
Alignment of the best matching sequences from wheat (top) and barley (bottom) (accessions ABR04074.1 and BAJ85510.1, respectively) in the NCBI nr protein database with the sequence of soybean lunasin. ABR04074.1 corresponds to HMW subunit 1By16 from *Triticum dicoccoides*.

## References

[bib1] Galvez A.F., de Lumen B.O. (1999). A soybean cDNA encoding a chromatin-binding peptide inhibits mitosis of mammalian cells. Nature Biotechnology.

[bib2] Hernández-Ledesma B., Hsieh C.-C., de Lumen B.O. (2009). Lunasin, a novel seed peptide for cancer prevention. Peptides.

[bib3] International Barley Sequencing Consortium (2012). A physical, genetic and functional sequence assembly of the barley genome. Nature.

[bib4] Jeong H.J., Lam Y., de Lumen B.O. (2002). Barley lunasin suppresses ras-induced colony formation and inhibits histone acetylation in mammalian cells. Journal of Agricultural and Food Chemistry.

[bib5] Jeong H.J., Park J.H., Lam Y., de Lumen B.O. (2003). Characterization of lunasin isolated from soybean. Journal of Agricultural and Food Chemistry.

[bib6] Jeong H.J., Jeong J.B., Kim D.S., Park J.H., Lee J.B., Kweon D.H., Chung G.Y., Seo E.W., de Lumen B.O. (2007). The cancer preventive peptide lunasin from wheat inhibits core histone acetylation. Cancer Letters.

[bib7] Jeong H.J., Lee J.R., Jeong J.B., Park J.H., Cheong Y.K., de Lumen B.O. (2009). The cancer preventative seed peptide lunasin from rye is bioavailable and bioactive. Nutrition and Cancer.

[bib8] Jeong H.J., Jeong J.B., Hsieh C.C., Hernández-Ledesma B., de Lumen B.O. (2010). Lunasin is prevalent in barley and is bioavailable and bioactive in *in vivo* and *in vitro* studies. Nutrition and Cancer.

[bib9] Jeong J.B., Jeong H.J., Park J.H., Lee S.H., Lee J.R., Lee H.K., Chung G.Y., Choi J.D., de Lumen B.O. (2007). Cancer-preventive peptide lunasin from *Solanum nigrum* L. inhibits acetylation of core histones H3 and H4 and phosphorylation of retinoblastoma protein (Rb). Journal of Agricultural and Food Chemistry.

[bib10] Lin J., Fido R., Shewry P., Archer D.B., Alcocer M.J.C. (2004). The expression and processing of two recombinant 2S albumins from soybean (*Glycine max*) in the yeast *Pichia pastoris*. Biochimica et Biophysica Acta.

[bib11] Maldonado-Cervantes E., Jeong H.J., León-Galván F., Barrera-Pacheco A., De León-Rodríguez A., Gonzales de Maija E., de Lumen B., Barba de la Rosa A.P. (2010). Amaranth lunasin-like peptide internalizes into the cell nucleus and inhibits chemical carcinogen-induced transformation of N1H-3T3 cells. Peptides.

[bib12] Monsalve R.I., Villalba M., Rico M., Shewry P.R., Rodriguez R., Mills E.N.C., Shewry P.R. (2003). The 2S albumin proteins. Plant Food Allergens.

[bib13] Nakurte I., Klavins K., Kirhnere I., Namniece J., Adlere L., Matvejevs J., Kronberga A., Kokare A., Strazdina V., Legzdina L., Muceniece R. (2012). Discovery of lunasin peptide in triticale (*X Triticosecale* Wittmack). Journal of Cereal Science.

[bib14] Odani S., Koide T., Ono T. (1987). Amino acid sequence of a soybean (Glycine max) seed polypeptide having a poly (L-aspartic acid) structure. Journal of Biological Chemistry.

[bib15] Pellny T.K., Lovegrove A., Freeman J., Tosi P., Love C.G., Knox J.P., Shewry P.R., Mitchell R.A.C. (2012). Cell walls of developing wheat starchy endosperm: comparison of composition and RNA-Seq transcriptome. Plant Physiology.

[bib16] Revilleza M.J., Galvez A.F., Krenz D.C., de Lumen B.O. (1996). An 8 kDa methionine-rich protein from soybean (*Glycine max*) cotyledon: identification, purification, and N-terminal sequence. Journal of Agricultural and Food Chemistry.

[bib17] Rizzello C.G., Nionelli L., Coda R., Gobbetti M. (2012). Synthesis of the cancer preventative peptide lunasin by lactic acid bacteria during sourdough fermentation. Nutrition and Cancer.

[bib18] Shewry P.R., Jenkins J., Beaudoin F., Mills E.N.C., Mills E.N.C., Shewry P.R. (2004). The classification, functions and evolutionary relationships of plant proteins in relation to food allergens. Plant Food Allergens.

[bib19] Shewry P.R., Pandya M.J., Shewry P.R., Casey R. (1999). The 2S albumins storage proteins. Seed Proteins.

[bib20] Silva-Sánchez C., de la Rosa A.P., León-Galván M.F., de Lumen B.O., de León-Rodríguez A., de Majía E.G. (2008). Bioactive peptides in amaranth (*Amaranthus hypochondriacus*) seed. Journal of Agricultural and Food Chemistry.

